# Association between culture and the preference for, and perceptions of, 11 routes of medicine administration: A survey in 21 countries and regions

**DOI:** 10.1016/j.rcsop.2023.100378

**Published:** 2023-11-26

**Authors:** Sudaxshina Murdan, Li Wei, Diana A. van Riet-Nales, Abyot Endale Gurmu, Stella Folajole Usifoh, Adriana-Elena Tăerel, Ayca Yıldız-Peköz, Dušanka Krajnović, Lilian M. Azzopardi, Tina Brock, Ana I. Fernandes, André Luis Souza dos Santos, Berko Panyin Anto, Thibault Vallet, Eunkyung Euni Lee, Kyeong Hye Jeong, Marwan Akel, Eliza Tam, Daisy Volmer, Tawfik Douss, Sharvari Shukla, Shigeo Yamamura, Xiaoe Lou, Bauke H.G. van Riet, Cyril O. Usifoh, Mahama Duwiejua, Fabrice Ruiz, Adrian Furnham

**Affiliations:** a UCL School of Pharmacy, 29-39 Brunswick Square, London WC1N 1AX, UK; b Medicines Evaluation Board Utrecht, the Netherlands; c Department of Pharmacognosy, School of Pharmacy, College of Medicine and Health Sciences, University of Gondar, Gondar, Ethiopia; d Department of Clinical Pharmacy & Pharmacy Practice, Faculty of Pharmacy, University of Benin, Benin City, Nigeria; e Faculty of Pharmacy, University of Medicine and Pharmacy “Carol Davila”, Bucharest, Romania; f Dept. of Pharmaceutical Technology, Faculty of Pharmacy, Istanbul University, Turkey; g Department of Social Pharmacy and Pharmaceutical Legislation, Faculty of Pharmacy, University of Belgrade, Belgrade, Serbia; h Department of Pharmacy, Faculty of Medicine and Surgery University of Malta, Msida, Malta; i Department of Clinical Pharmacy, UCSF School of Pharmacy, UCSF Box 0622, 521 Parnassus Ave, San Francisco 94143, USA; j Egas Moniz Center for Interdisciplinary Research (CiiEM), Egas Moniz School of Health & Science, 2829-511 Caparica, Almada, Portugal; k Department of General Microbiology, Federal University of Rio de Janeiro (UFRJ), Rio de Janeiro, Brazil; l Dept of Clinical & Social Pharmacy, Faculty of Pharmacy & Pharmaceutical sciences, KNUST, Kumasi, Ghana; m ClinSearch, 110 Avenue Pierre Brossolette, 92240 Malakoff, France; n Seoul National University College of Pharmacy, Republic of Korea; o College of Pharmacy, Chung-Ang University, Republic of Korea; p School of Pharmacy, Lebanese International University, Lebanon; q Department of Pharmacology and Pharmacy, The University of Hong Kong, Hong Kong; r Institute of Pharmacy, Faculty of Medicine, University of Tartu, 50411 Tartu, Estonia; s Faculty of Pharmacy of Monastir, Tunisia; t Symbiosis Statistical Institute, Symbiosis International (Deemed University), Pune, India; u Department of Biostatistics, Faculty of Pharmaceutical Sciences, Josai International University, Togane, Chiba, Japan; v Zhejiang University, China; w Department of Radiotherapy, Netherlands Cancer Institute (at the time of participant recruitment in the Netherlands: MEB and VU University Medical Center), Amsterdam, the Netherlands; x Dept Leadership and Orgnaisational Behaviour, Norwegian Business School, Norway; y Inspect-Lb (Institut National De Santé Publique, D'épidémiologie Clinique Et De Toxicologie-Liban), Beirut, Lebanon

**Keywords:** Medicine, Route of administration, Culture, Perception, Preference, Association

## Abstract

Medicines can be taken by various routes of administration. These can impact the effects and perceptions of medicines. The literature about individuals' preferences for and perceptions of the different routes of administration is sparse, but indicates a potential influence of culture. Our aim was to determine: (i) any association between one's culture and one's preferred route of medicine administration and (ii) individual perceptions of pain, efficacy, speed of action and acceptability when medicines are swallowed or placed in the mouth, under the tongue, in the nose, eye, ear, lungs, rectum, vagina, on the skin, or areinjected.

A cross-sectional, questionnaire-based survey of adults was conducted in 21 countries and regions of the world, namely, Tunisia, Ghana, Nigeria, Turkey, Ethiopia, Lebanon, Malta, Brazil, Great Britain, United States, India, Serbia, Romania, Portugal, France, Netherlands, Japan, South Korea, Hong Kong, mainland China and Estonia, using the Inglehart–Welzel cultural map to ensure coverage across all cultures. Participants scored the pain/discomfort, efficacy, speed of onset and acceptability of the different routes of medicine administration and stated their preferred route. Demographic information was collected.

A total of 4435 participants took part in the survey. Overall, the oral route was the most preferred route, followed by injection, while the rectal route was the least preferred. While the oral route was the most preferred in all cultures, the percentage of participants selecting this route varied, from 98% in Protestant Europe to 50% in the African-Islamic culture. A multinomial logistic regression model revealed a number of predictors for the preferred route. Injections were favoured in the Baltic, South Asia, Latin America and African-Islamic cultures while dermal administration was favoured in Catholic Europe, Baltic and Latin America cultures. A marked association was found between culture and the preference for, and perceptions of the different routes by which medicines are taken. This applied to even the least favoured routes (vaginal and rectal). Only women were asked about the vaginal route, and our data shows that the vaginal route was slightly more popular than the rectal one.

## Highlights

• There is an association between culture and preference/perceptions of medication route.
• Overall, the oral route was the most preferred route, followed by injection.
• The dominance of the oral route varied by culture.
• Injections are favoured in Baltic, South Asia, Latin America and African-Islamic.
• Even the least preferred routes, rectal and vaginal, had a cultural dimension.

## 1 Introduction

Medicines are most often taken by the oral route of administration,^1^ and it is commonly believed that most people prefer to take medicines this way.^2^ It is also commonly assumed that most people would prefer to avoid the injection route if they had a choice.^2^ There is however, evidence that in some countries, injections are preferred over other routes of administration.^3^^,^^4^ The rectal route is unthinkable in some countries, somewhat better accepted in others and well accepted in a few countries for certain age groups or indications, e.g. paracetamol suppositories for infants.^5^^,^^6^ In evidencing these assumptions however, the literature and international regulatory guidance is sparse regarding people's perceptions of the different routes by which medicines can be taken. For example, there is much more research about the (lower) acceptability of injections than about other routes of administration. This may be due to the local pain and/or visceral reaction to injections felt by many individuals. It may also be related to the greater use of injections in pharmacotherapy compared to certain other routes of administration, such as vaginal and otic. When more than one route of medicine administration has been investigated with respect to patients' preference, the oral and injection routes have most often been compared to each other.^7^, ^8^, 9., 10 Other commonly-used routes of administration, such as ocular, have been less investigated. Moreover, the severely unequal geographical distribution of investigations on patient acceptability of medicines means that bias is inherent in the open literature. Yet the commonly-held beliefs, anecdotal evidence and literature referenced above indicates that the preference for, and perception of the different routes by which medicines can be taken, may be influenced by an individual's culture.

The term ‘culture’ has multiple meanings in different disciplines and contexts. For example, it is often used to refer to the intellectual, musical, artistic and literary products of a society, while anthropologists have used it to refer to a society's values, practices, symbols, institutions and human relationships.^11^^,^^12^ In this paper, culture is defined as the ideas, customs and social behaviour of a particular people or society. Culture is known to partly shape health attributions, health beliefs and health behaviours, leading to a diversity in belief systems about healing among cultural groups.^13^ Cultural background also informs beliefs about medication, such as beliefs about the benefits and dangers of medicines,^14^ drug efficacy,^15^ and affects behaviours, such as, adherence to medication^15^, ^16^, ^17^ including prescription drugs.^18^ Religious concerns has had a long history^19^ and can lead to discontinuation of medication, when medicine components are considered prohibited,^20^ such as certain animal-derived products,^21^ or during observances such as the Ramadan fasting period.^22^ The literature also shows that relationships are not always clear-cut, with negative, null or positive associations between religiosity and medication adherence having been reported.^23^^,^^24^

The aim of the work described in this paper was to address the lack of data on people's beliefs about the various routes of medicine administration, and determine any association between an individual's culture and their preferred route of medicine administration and perceptions of the pain/discomfort, efficacy, speed of action and acceptability of the following commonly-used routes: oral, buccal, sublingual, nasal, ocular, otic, pulmonary/inhalation, parenteral, dermal (topical on the skin), rectal and vaginal.

## 2 Methods

### 2.1 Selection of countries where the research was to be conducted

**Fig. 1:**
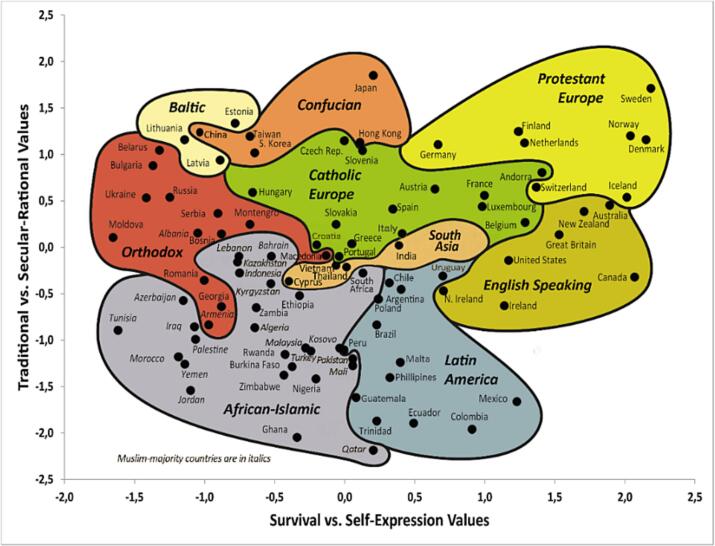
Inglehart–Welzel cultural map of the world (Wave 6; 2010–2014 used in this study) shows where countries are located on two major dimensions of cross-cultural variation in the world, namely, (i) traditional values versus secular-rational values and (ii) survival values versus self-expression values. From.^26^

A questionnaire-based, cross-sectional study was performed in different cultural regions of the world. To enable the selection of countries and regions where the survey was to be conducted, the Inglehart–Welzel (IW) cultural map of the world (Fig. 1) produced by the World Values Survey (WVS) was used. In this map, countries are grouped into culture clusters, based on people's values, beliefs and norms. The map is based on two major dimensions of cross-cultural variation, namely, (i) how important a role religious doctrine plays in societies and (ii) how autonomous from kinship obligations individuals in a society are in their life planning.^26^ The IW map was selected for several reasons: it is produced by the WVS, a project implemented by scientists from 120 countries and societies, meaning its coverage is broad and local input is assured. It is also the map which is the most widely used for cross-national survey by scientists, governments and international organisations.^25^ In this study, IW map from Wave 6 (2020–2014) of the World Values Survey^25^ was used, as this was the up-to-date version at the time the study was planned and initiated. The WVS Wave 6 grouped countries into nine culture clusters, namely African-Islamic, Latin America, English Speaking, South Asia, Orthodox, Catholic Europe, Protestant Europe, Confucian and Baltic as shown in Fig. 1.

Principal investigators (PIs) from a number of countries and regions were invited to take part in the study through a network of colleagues, ensuring that all the nine culture regions of the IW map were represented. The countries and regions by culture were as follows: African-Islamic (Tunisia, Ghana, Nigeria, Turkey, Ethiopia, Lebanon), Latin America (Malta, Brazil), English Speaking (Great Britain, United States), South Asia (India), Orthodox (Serbia, Romania), Catholic Europe (Portugal, France), Protestant Europe (Netherlands), Confucian (Japan, South Korea, Hong Kong and mainland China) and Baltic (Estonia). While the number of countries per culture region was not predefined, it was ensured that the survey would take place in at least one country for each culture region. In addition, careful attention was paid to ensure that all six WHO Regions (Africa, Americas, South-East Asia, Europe, Eastern Mediterranean, Western Pacific), and all income classes (low, lower middle, upper middle and high, as classified by the World Bank) were included.

### 2.2 Questionnaire development

The survey questionnaire, was drafted by the UK team (UCL Ethics Project ID Number: CEHP/2012/035), and revised following input from principal investigators in other countries to reflect local realities and results from any pre-testing of the questionnaire, such as which religions to include and the terminology. Once a master questionnaire was agreed upon, it was sent to each local PI who translated the questionaire into the local language (in all countries apart from the UK and USA), and organised ethics/Institutional Review Board approval and data collection. The questionnaire was translated into the following languages: Arabic in Tunisia and Lebanon; Yoruba, Hausa and Ibo in Nigeria; Turkish in Turkey; Amharic in Ethiopia; Maltese in Malta; Portuguese in Brazil and Portugal; Marathi in India (in Pune); Serbian in Serbia; Romanian in Romania; French in France; Dutch in the Netherlands; Japanese in Japan; Korean in South Korea, Chinese in Hong Kong and mainland China and Estonian in Estonia. The questionnaire may show minor differences upon translation for regional requirements, however the the master questionnaire (Supplementary Form A) remained the same.

The questionaire started with a very brief introduction and explanation to summarize the purpose of the study to participants, which was followed by two sections. The first section had 45 questions and asked participants to score, on a scale of 1–10, the pain/discomfort, efficacy, speed of action and acceptability of 11 routes of medicine administration and asked them to state which one route they would choose, if given the option. The second section comprised 11 questions and asked participants about their characteristics in order to determine whether any of these characteristics might be associated with an individual's perception of the different routes of medicine administration.

The 11 routes included in the first section were: oral, buccal, sublingual, nasal, ocular, otic, pulmonary, parenteral, dermal, rectal and, for females, also vaginal. The participant information requested in the second section included their year of birth, sex, residence (city, town, village, countryside, other), ethnicity (European, Asian, African, Arabic, Chinese, Korean, other), religion (Christian, Muslim, Buddhist, Hindu, Jewish, African traditional religion, none, other), how religious they were (on a scale of 1–10, where 1 indicates not religious, and 10 very religious), their highest educational qualification (none, primary school, high school, technical qualification, undergraduate university degree, postgraduate university degree, other), occupation, how wealthy they considered themselves to be in their country (very rich, rich, average, poor, very poor), how they would describe their health (on a scale of 1 to 10, where 10 indicates the best), and if they were taking any medicines at present.

### 2.3 Data collection

Local PIs were asked to collect data from about 200 participants from the general public, and the sampling method was left to the national researcher for feasibility and pragmatic reasons. Convenience sampling, a non-probability sampling method where units are selected for inclusion in the sample because they are the easiest for the researcher to access, possibly due to geographical proximity, availability at a given time, or willingness to participate in the research, was used, and participants were recruited at various locations. A sample size of 200 participants per country was based on typical sample sizes used in similar surveys.

Data was collected in person in every country/region with two exceptions: all responses were collected online in Hong Kong while in Malta, data was collected in-person and online. Whether data collection took place online or in-person was decided by local PIs depending on their capacity. The venues for the in-person data collection included pharmacies, markets, homes, coffee shops, town centre, workplaces, shops, and in and around metro, train and taxi stations. Online data collection took place using Qualtrics (Provo, Utah, USA). Data collection took place from 2016 (e.g. in China, Republic of Korea) until 2020 (Brazil). This long duration occurred due to the time it took to recruit PIs from a range of countries to ensure sufficient coverage in all the nine clusters of the IW cultural map, and where needed, to obtain additional national ethics/institutional Review Board approval.

Responses were captured by each PI and entered into a standardised Microsoft Excel spreadsheet and sent to the corresponding author, who collated and coded all the data, and prepared the masterfile for analysis by SPSS. In case of data irregularities, clarification was sought from the local PI.

### 2.4 Data analysis

Data were anaysed with respect to the following variables: route of administration, participant's age, gender, residence, ethnicity, religion, religiosity, education, wealth, health and if participants are taking any medicines. The study outcomes were perceived pain/discomfort, efficacy, speed of action, acceptability of the different routes of medicine administration and preferred route for taking medicines.

### 2.5 Statistical analyses

Data are presented as mean (standard deviation - SD) or median (interquartile range – IQR), for the continuous variables and number (percentage - %) for the categorical variables. One-way Analysis of variance (ANOVA) and post hoc Bonferroni test was conducted to test the score difference for the perceptions of the pain/discomfort, efficacy, speed of action and acceptability among the different routes. The factors associated with the scores of the perceptions of the pain/discomfort, efficacy, speed of action and acceptability were analysed by linear regression. A multinomial logistic regression model with 95% confidence interval (CI) was employed to study the association between the individual's culture and the preferred route of medicine administration. Repeated measures ANOVA and post hoc Bonferroni was used to compare the different routes with regards to the pain/discomfort, efficacy, speed of action and acceptability for respondents with complete datasets. Paired *t-*tests were conducted to compare the females' scores for the rectal and vaginal routes. A two-sided *p*-value of <0.05 was used to identify statistically significant results. Statistical software of SPSS (version 27; Chicago, US) was used to analyse the data.

### 2.6 Role of the funding source

The funders did not have any role in study design, data collection, analysis, and interpretation, the writing of the report; and the decision to submit the paper for publication.

## 3 Results

### 3.1 Demographics

The data is shown in Table 1. Altogether the questionnaire survey was completed by 4435 adults, aged 18 to 94 years, with slightly more females than males. The median number of participants per country/region was 201, with the fewest in Hong Kong and the greatest in Nigeria. The large number of participants in Nigeria was due to data collection in Nigeria's 3 main dialects, Yoruba, Ibo and Hausa, without a corresponding reduction of the intended ∼200 participants per country. Considering the IW culture clusters, the proportions of participants per cluster ranged from 5% (in Protestant Europe, Baltic and South Asia) to 33% (in African-Islamic). The proportions of participants who lived in countries at different income levels ranged from 4% (low income) to 39% (in high income). With respect to WHO Region, the proportions of participants per region ranged from 5% (South East Asia) to 42% (European). The majority of participants lived in urban areas. Participants were of diverse ethnicities and religions, including of no religion. Education levels ranged from university or technical college qualifications (the majority of participants) to no formal schooling (a small proportion). Slightly more than half of respondents were not taking any medicines. The mean score for how participants considered their health and religiosity was 7.3 and 5.3 respectively. Most respondents scored themselves as average regarding wealth. Participants' occupation was diverse, and although this was asked for, this item was excluded from data analysis due to the large diversity. The amount of missing data was low, both for responses about the routes of medicine administration (3%) and demographics (3%).

**Table 1:** Demographics data.

Number of respondents	4435 (56% females; 44% males), aged 18 and 94 years old, with a median age of 32 years (IQR 25–48).
Method of survey	98% in-person, 2% online.
Number of respondents per country	mean = 211; mode = 200; median = 201; minimum of 66 in Hong Kong and maximum of 497 in Nigeria
Countries in the different culture clusters in the IW cultural map	African-Islamic: Tunisia, Ghana, Nigeria, Turkey, Ethiopia, Lebanon
	Latin America: Malta, Brazil
	English Speaking: Great Britain, United States
	South Asia: India
	Orthodox: Serbia, Romania
	Catholic Europe: Portugal, France
	Protestant Europe: Netherlands
	Confucian: Japan, Hong Kong, South Korea, China
	Baltic: Estonia
% of respondents who lived in the following culture clusters in the IW cultural map	Confucian: 15%
	Protestant Europe: 5%
	English Speaking: 9%
	Catholic Europe: 10%
	Orthodox: 9%
	Baltic: 5%
	South Asia: 5%
	Latin America: 10%
	African-Islamic: 33%
% of respondents who lived in countries with the following income levels (as defined by the World Bank)	low income:4%
	lower middle: 29%
	upper middle: 28%
	high income: 39%
% of respondents who lived in the following WHO Region	African: 20%
	Americas: 9%
	South East Asia: 5%
	European: 42%
	Eastern Mediterranean: 9%
	Western Pacific: 15%
Respondents' residence	16% in rural; 79% in urban areas
Respondents' ethnicity	40% European; 22% African; 13% Asian, 9% Arabian, 5% Chinese, 5% Korean, 5% Turkish, 2% Other, 0.7% Mixed, 0.1% Caribbean, 0.1% American.
Respondents' religion⁎	None (24%), Christians (48%), Muslim (17%), Hindu (4%), Buddhist (4%), Other (1%), Traditional African (0.7%), Sikh (0.1%), Jewish (0.1%), Agnostic (0.1%), Spiritual (0.1%), Jain (0.02%)
Highest education level	56% had university or technical college qualifications, 26% had completed high school, 3% middle school, 6% primary school and 4% had no formal schooling.
Were respondents taking any medicine?	54% no; 45% yes
Participants' health	mean = 7.3 (with 10 being highest possible score)
Participants' religiosity	mean = 5.3 (with 10 being highest possible score)
Participants' wealth	1% very poor; 12% poor; 71% average; 13% rich; 2% very rich
Participants' occupation	Participants had a very large diversity of occupations, and although this was asked for, this item was excluded from data analysis due to the large diversity.
Missing data	3% for participants' responses about the routes of drug administration and 3% for covariates (participants' year of birth, gender, rural or urban residence, ethnicity, religion and how religious they were, highest education level, wealth, health and if they were taking any medicine.

⁎ All denominations of a religion were grouped, for example, the 48% Christians include Protestants, Catholics, Orthodox Christians and any other denominations.

### 3.2 Preferred route for taking medicines

**Fig. 2:**
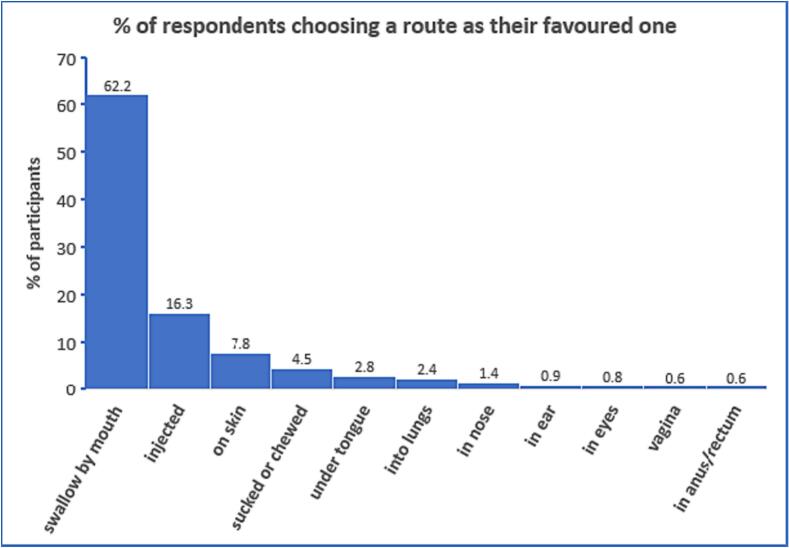
Percentage of respondents who would choose a particular route if offered the choice (*N* = 4354 males and females for all routes; except for vaginal, where *N* = 2424 as only females' responses were sought). The oral route was, by far, the preferred route for taking medicines, followed by, in order, injection, dermal, buccal, sublingual, inhalation, nasal, otic, ocular, vaginal (females only) and rectal.

The majority of participants (62%) chose the oral route as the preferred route for taking medicines (Fig. 2). Injection was in second place, with 16% of the participants preferring this route of administration. Dermal was in third place, followed by buccal, sublingual, pulmonary, nasal, otic and ocular, while the rectal route was chosen by the fewest number of participants (0.6%). When the data was analysed separately for males and females, the trends were broadly similar as that shown in Fig. 2. Females were also asked about the vaginal route, and this was slightly preferred to the rectal route (0.6% versus 0.5% of participants).

### 3.3 Association between culture and preferred route for taking medicines

**Fig. 3:**
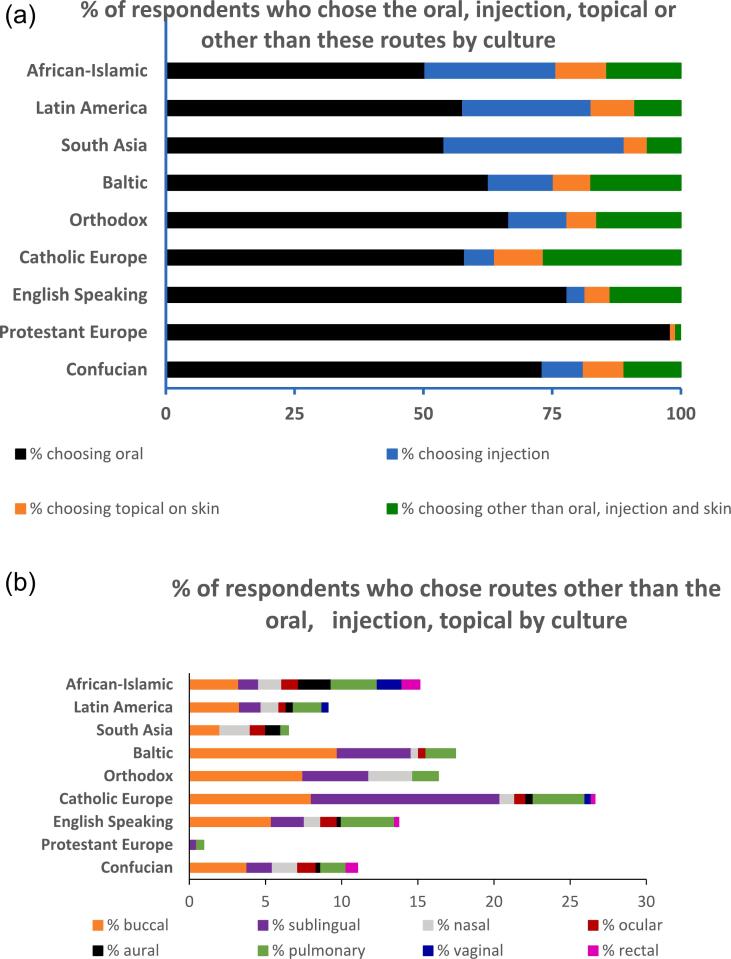
a: Percentage of respondents who choose the oral, parenteral, dermal (topical on the skin) or other routes as their preferred option by culture. The buccal, sublingual, pulmonary, nasal, otic, ocular, vaginal and rectal routes have been grouped under ‘other’ in this figure for ease of visualisation and are shown in Fig. 3b. *N* = 4435. This figure shows cultural differences in favoured route. For example, although the oral route was chosen by the majority of participants, its popularity varied by culture, being highest in Protestant Europe and lowest in African-Islamic culture. Similarly, while the injection route was not preferred by any participant in Protestant Europe, a third of South Asian, a quarter of Latin American, a quarter of African-Islamic and 13% of Baltic participants preferred this route. b: Percentage of respondents who would choose routes other than the oral, injection and dermal as the preferred route, namely the buccal, sublingual, pulmonary, nasal, otic (aural), ocular, vaginal and rectal routes by culture (N = 4354 for all routes; except for vaginal, where N = 2424 as only females' responses were considered). This figure shows the diversity of routes preferred by participants in different cultures. For example, in Catholic Europe, more than a quarter of participants preferred routes other than the oral, injection and dermal, in contrast to Protestant Europe where few participants (1%) did so.

When the preferred route was analysed by culture, notable differences were found (Fig. 3a-b; Table 2). For example, while the oral route was selected by 98% of participants in Protestant Europe, only 50% of participants in the African-Islamic culture selected this route as their preferred route (Fig. 3a). The parenteral route was chosen by 35% of participants in South Asia, 25% of participants in Latin America and in African-Islamic cultures, 13% of participants in the Baltic and 0% of participants in Protestant Europe (Fig. 3a). The preference for the buccal, sublingual, pulmonary, nasal, otic, ocular, vaginal and rectal routes of administration was also associated with culture (Fig. 3b). For example, a larger percentage of participants in Catholic Europe chose the sublingual route, compared to other cultures. The rectal and vaginal routes were chosen by 1–2% of participants in African-Islamic culture, and by none in most of the other cultures. Fig. 3a and b also show that there was greater diversity in the preferred route in some cultures, e.g. in Catholic Europe compared to others, e.g. protestant Europe.

**Table 2:** % of respondents in each culture who selected each of these routes as their preferred option. N = 4354 for all routes, except for vaginal where N = 2424 as only females were asked about the vaginal.

	% respondents choosing each route as preferred option by culture										
Culture	Oral	Injection	Dermal	Buccal	Sublingual	Nasal	Ocular	Otic	Pulmonary	Vaginal	Rectal
Confucian	73.1	8.0	7.9	3.8	1.7	1.7	1.2	0.3	1.7	0.0	0.8
Protestant Europe	98.0	0.0	1.0	0.0	0.5	0.0	0.0	0.0	0.5	0.0	0.0
English Speaking	77.9	3.5	4.9	5.4	2.2	1.1	1.1	0.3	3.5	0.0	0.3
Catholic Europe	58.0	5.8	9.5	8.0	12.4	1.0	0.7	0.5	3.4	0.4	0.2
Orthodox	66.6	11.3	5.8	7.5	4.3	2.9	0.0	0.0	1.7	0.0	0.0
Baltic	62.6	12.6	7.3	9.7	4.9	0.5	0.5	0.0	1.9	0.0	0.0
South Asia	54.0	35.0	4.5	2.0	0.0	2.0	1.0	1.0	0.5	0.0	0.0
Latin America	57.6	24.9	8.5	3.3	1.4	1.2	0.5	0.5	1.9	0.4	0.0
African-Islamic	50.3	25.4	9.9	3.2	1.3	1.5	1.1	2.1	3.0	1.6	1.2

To investigate the association between culture and other related/unrelated factors on participants' preferred route in more detail, the less preferred buccal, sublingual, pulmonary, nasal, otic, ocular, vaginal and rectal routes were grouped into ‘other’ and a multinomial logistic regression analysis was conducted for the injection and dermal routes, using the oral route as the reference group. The multinomial logistic regression model revealed a number of predictors for the choice of a route as the preferred option (Table 3). Injections are favoured in the Baltic, South Asia, Latin America and African-Islamic cultures, and those who have a medium education level, who are poor, and are of African ethnicity. Dermal administration is favoured in Catholic Europe, Baltic and Latin America cultures, and those who are younger, female, and of African and Arabian ethnicities.

**Table 3:** Predictors of which route was selected as the preferred route, following multinomial logistic regression analysis and using the oral route as reference (odds ratio and confidence intervals are shown, p < 0.05).

	Injection			Dermal			Others		
	OR	95%CI		OR	95%CI		OR	95%CI	
Age (+1)	1.006	0.999	1.012	**0.987**	**0.979**	**0.996**	1.004	0.995	1.013
Gender (female vs male)	0.965	0.806	1.155	**1.615**	**1.266**	**2.059**	1.098	0.851	1.417

Education									
postgraduate	1			1			1		
none	0.915	0.527	1.587	0.600	0.256	1.405	0.641	0.305	1.351
primary	1.345	0.846	2.136	1.035	0.550	1.948	1.121	0.625	2.012
middle	**2.651**	**1.452**	**4.840**	1.665	0.713	3.887	0.877	0.262	2.933
high	1.405	0.963	2.050	1.061	0.679	1.658	0.807	0.512	1.272
technical	0.889	0.578	1.369	1.140	0.693	1.875	0.804	0.479	1.350
undergraduate	1.292	0.889	1.878	1.147	0.748	1.758	0.721	0.464	1.120
other	2.013	0.954	4.248	1.262	0.400	3.980	0.993	0.344	2.868

Wealth									
very rich	1			1			1		
very poor	2.301	0.902	5.868	3.036	0.787	11.718	**3.417**	**1.063**	**10.982**
poor	**1.817**	**1.020**	**3.236**	1.725	0.641	4.644	1.166	0.513	2.650
average	1.408	0.822	2.412	1.941	0.760	4.959	1.286	0.612	2.702
rich	1.170	0.657	2.085	1.988	0.752	5.254	1.056	0.479	2.328

Rural/Urban									
Urban	1			1			1		
Rural	0.764	0.583	1.002	1.287	0.935	1.772	1.383	0.981	1.951

Religion									
none	1			1			1		
Buddhist	0.938	0.474	1.855	1.355	0.748	2.454	0.953	0.476	1.906
Christian	0.858	0.599	1.230	0.637	0.428	0.947	0.810	0.511	1.284
Muslim	1.452	0.904	2.332	0.757	0.416	1.376	0.543	0.279	1.059
Hindu	0.302	0.088	1.030	0.590	0.055	6.352	0.663	0.045	9.661
Others	1.451	0.780	2.698	1.078	0.480	2.420	0.802	0.317	2.033

Ethnicity									
European	1			1			1		
African	**2.957**	**1.656**	**5.279**	**2.822**	**1.413**	**5.635**	1.814	0.694	4.742
Asian	0.885	0.309	2.529	1.496	0.584	3.833	1.459	0.597	3.568
Arabian	0.972	0.493	1.918	**3.518**	**1.569**	**7.888**	1.109	0.374	3.283
Chinese	0.754	0.237	2.399	0.896	0.309	2.600	0.432	0.130	1.434
Korean	2.406	0.774	7.481	1.682	0.554	5.104	1.447	0.474	4.417
Turkish	1.471	0.707	3.064	2.598	0.976	6.917	1.753	0.528	5.823
others	**2.174**	**1.257**	**3.761**	1.808	0.838	3.899	1.508	0.583	3.896

Culture									
English Speaking	1			1			1		
Confucian	2.096	0.800	5.494	1.717	0.788	3.742	0.897	0.405	1.988
Protestant Europe	–			0.372	0.099	1.405	**0.069**	**0.009**	**0.541**
Catholic Europe	2.078	0.951	4.539	**3.411**	**1.629**	**7.141**	1.082	0.537	2.177
Orthodox	1.295	0.515	3.255	1.199	0.423	3.395	0.382	0.126	1.156
Baltic	**4.737**	**2.114**	**10.616**	**3.040**	**1.264**	**7.314**	0.507	0.181	1.418
South Asia	**130.647**	**24.758**	**689.432**	1.439	0.105	19.781	0.552	0.032	9.560
Latin America	**6.967**	**3.368**	**14.413**	**3.130**	**1.421**	**6.896**	0.889	0.399	1.979
African-Islamic	**5.975**	**2.593**	**13.769**	1.980	0.801	4.892	2.226	0.771	6.431

### 3.4 The preferred route's pain/discomfort, efficacy, speed of action and acceptability scores

**Fig. 4:**
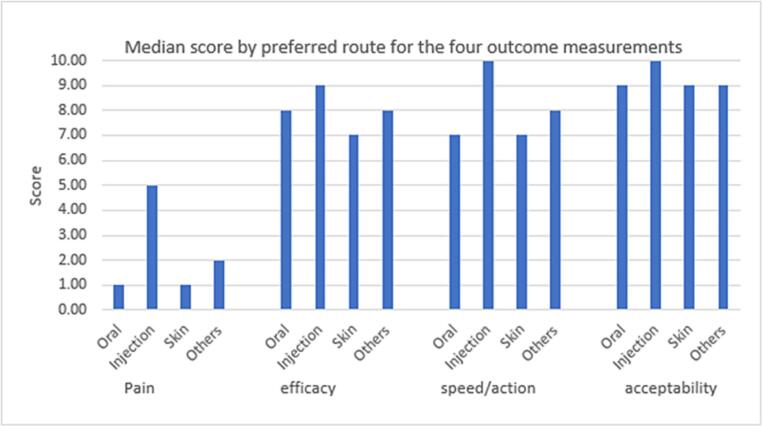
Median pain/discomfort, efficacy, speed of action and acceptability scores of the preferred routes of drug administration. Significant differences in pain/discomfort, efficacy, speed of action and acceptability scores of oral and injection routes were found (*p* < 0.05). Individuals who preferred the injection route perceived them to be more painful, more efficacious, more acceptable and have a faster speed of action than the oral route. ANOVA; Bonferroni post hoc test conducted separately for pain/discomfort, efficacy, speed of action and acceptability.

When the scores of the *preferred* routes of medicine administration were compared (Fig. 4), significant differences were found between the pain/discomfort, efficacy, speed of action and acceptability scores of the oral and parenteral routes (Bonferroni post hoc test conducted separately for pain/discomfort, efficacy, speed of action and acceptability, *p* < 0.05). For people who favoured the parenteral route, injections were perceived to be more painful, more efficacious, have a faster speed of action, and be more acceptable than the oral route.

### 3.5 Perception of the pain/discomfort, efficacy, speed of action and acceptability of all routes investigated

**Fig. 5:**
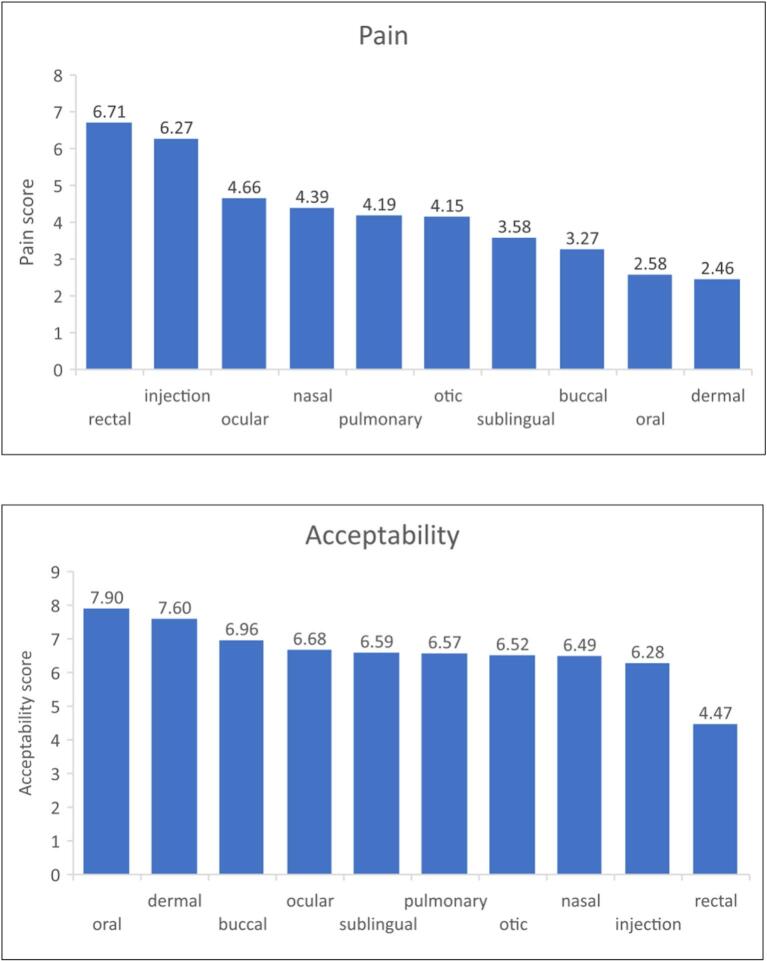

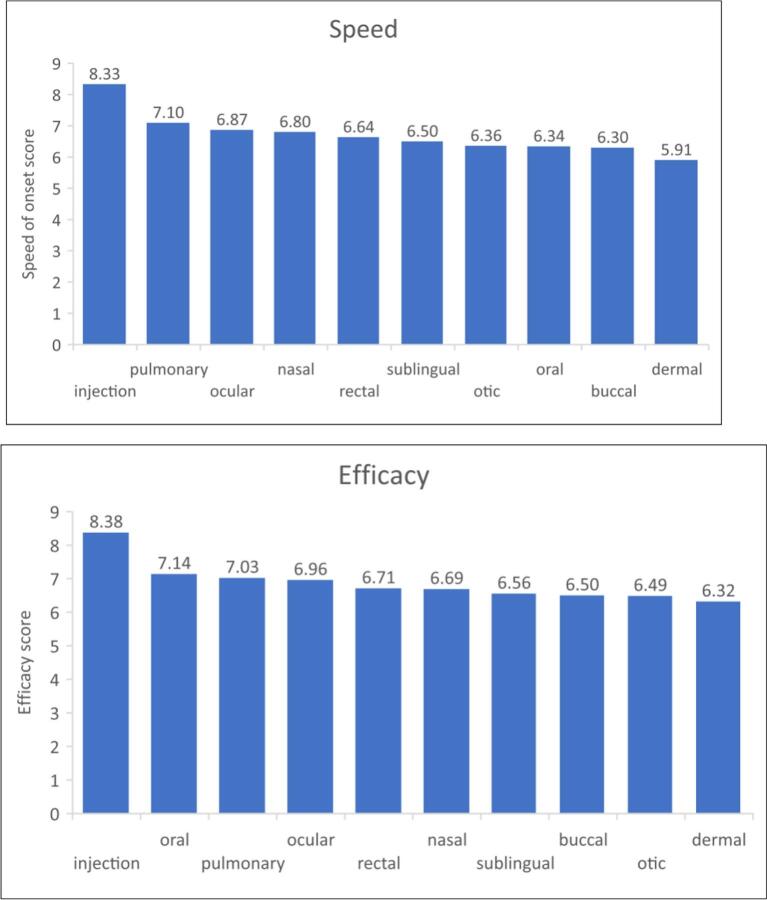
Mean scores for pain, acceptability, speed of onset and efficacy of the different routes of medicine administration for respondents with complete sets of scores (*N* = 4156 for pain; *N* = 4128 for efficacy; *N* = 4126 for speed of onset; *N* = 4144 for acceptability). One-way repeated measures ANOVA and Bonferroni test conducted to compare pain, efficacy, speed of action and acceptability scores of the different routes (excluding the vaginal route as males were not asked about this route).

The mean scores for pain/discomfort, efficacy, speed of action and acceptability, of all the routes of medicine administration (i.e., not just those that were selected as preferred) are shown in Table 4, for respondents (>93% of total study participants) with complete sets of scores. One-way repeated measures ANOVA and Bonferroni test conducted to compare pain, efficacy, speed of action and acceptability scores of the different routes (excluding vagina as males were not asked about this route) showed the following (p < 0.05). The rectal route was perceived to be the most painful/uncomfortable, even more so than injection, while the oral and dermal routes were perceived to be the least painful. The oral route had the highest acceptability scores, while the rectal route had the lowest. Injections were perceived as most efficacious and to have the fastest onset of action with dermal being the opposite (i.e. least efficacious and slowest) (Fig. 5). Although there was some variability, trends from lowest to highest scores for the different routes of administration were not affected by gender, culture, country income, WHO Region, and participants' religion, ethnicity and whether the participants were taking any medicines. When female participants' rectal and vaginal scores were compared, paired *t-*test showed the vaginal route to be perceived as less painful (score 6.2 vs 6.9, *p* < 0.0005) and more acceptable (score 5.0 vs 4.5, p < 0.0005), but having a slower onset of action (score 6.6 vs 6.8, p < 0.0005) and the same efficacy (score 6.8 vs 6.9, *p* = 0.2) when compared to the rectal route, although the absolute differences were small.

**Table 4:** Mean scores for perceived pain/discomfort, efficacy, speed of action and acceptability of the different routes. N = number of respondents for whom complete sets of scores were available, without any missing values. Participants were asked to score their answers from 1 (low) to 10 (high).

	Mean scores (SD)			
Route of administration	Pain	Efficacy	Speed	Acceptability
	N = 4156	N = 4128	N = 4126	N = 4144
	(*N* = 2131 for vaginal)	(*N* = 2115 for vaginal)	(*N* = 2114 for vaginal)	(*N* = 2128 for vaginal)
Oral	2.58 (2.39)	7.14 (2.10)	6.34 (2.18)	7.90 (2.51)
Buccal	3.27 (2.83)	6.50 (2.27)	6.30 (2.26)	6.96 (2.89)
Sublingual	3.58 (2.81)	6.56 (2.34)	6.50 (2.35)	6.59 (2.91)
Nasal	4.39 (2.86)	6.69 (2.25)	6.80 (2.22)	6.49 (2.83)
Ocular	4.66 (2.85)	6.96 (2.15)	6.87 (2.09)	6.68 (2.66)
Otic	4.15 (2.81)	6.49 (2.22)	6.36 (2.17)	6.52 (2.67)
Pulmonary	4.19 (2.88)	7.03 (2.29)	7.10 (2.28)	6.57 (2.80)
Injection	6.27 (3.07)	8.38 (1.97)	8.33 (1.99)	6.28 (3.10)
Dermal	2.46 (2.37)	6.32 (2.26)	5.91 (2.33)	7.60 (2.52)
Rectal	6.71 (3.08)	6.71 (2.37)	6.64 (2.36)	4.47 (3.05)
Vaginal (only females were asked about this route)	6.25 (3.16)	6.80 (2.30)	6.55 (2.36)	4.97 (3.17)

## 4 Discussion

This study in 21 countries and regions in all the zones of the IW Culture map and WHO Regions is the first study to include 11 routes of medicine administration, and to investigate whether there is an association between an individual's culture and their perceptions of, and preference for a route of medicine administration. The survey indicated that the oral route of administration was the most preferred, followed by injection, while the rectal and vaginal routes were the least preferred. The survey also showed a significant association between culture and the acceptability of the different medicine administration routes.

### 4.1 Association between culture and the most preferred route

The majority of participants preferred the oral route for taking medicines. This is in agreement with the existing literature, most of which indicates a preference for the oral route compared to injections e.g.^7^, ^8^, 9. with some exceptions e.g.^10^ The ease, convenience, and relative painlessness of the oral route leads to its prime position across all cultures, although it cannot be ruled out that this prime position may also relate to the prime use of this route of administration in pharmacotherapy. However, the preference for the oral route of administration was not uniform and was considerably higher in some cultures, such as in Protestant Europe and was lower in others such as the African-Islamic culture where routes other than oral were favoured by half of the participants. Culture is known to partly shape health attributions, health beliefs and health behaviours,^13^ as well as beliefs about medication,^14^ so it is not surprising that this study found an association between culture and participants' preferences for the different routes of medicine administration.

### 4.2 Culture and the least preferred routes of medicine administration (rectal and vaginal)

Even the least favoured vaginal and rectal routes showed a cultural dimension; these routes were chosen by 1–2% of participants in African-Islamic culture, and by none in most of the other cultures. This reflects findings by others that the rectal route is favoured in some countries and unthinkable in others.^5^ The unpopularity of the rectal and vaginal routes is reflected in the market size of rectal and vaginal formulations which make up <1% of the total pharmaceutical market.^26^ Nevertheless, it has to be acknowledged that even when the rectal and vaginal route may not be preferred, these routes are considered acceptable for certain age groups (such as for children) or indications (such as for local action, e.g. for vaginal infections), and are therefore included in national prescription guidelines and are considered acceptable in regulation without an actual study confirming adequate patient acceptance in the region where the product is to be marketed.

### 4.3 Association between culture and the injection route

The unexpected finding in this study was the second place taken by injection for preferred route in terms of % of participants. This seems to be in contrast to much research reporting that the fear of needles lead to many individuals avoiding medical treatment and immunization,^27^ for example, 27% of hospital employees in the USA avoided influenza vaccination because of their fear of the needle.^28^ Blood-injection-injury fears are also thought to explain approximately 10% of cases of COVID-19 vaccine hesitancy.^29^ At the same time, qualitative research in the 2000s found widespread popularity of injections in low and middle income countries and their excessive, unnecessary and unsafe administration by formal and informal providers, traditional healers and lay people.^3^ A recent paper also reported individuals' preference for injections over pills in Guinea-Bissau, Central African Republic, Democratic Republic of Congo and Sudan, due to beliefs about injections being ‘a real treatment’ and having a faster effect and greater potency.^4^ Our study confirms that the popularity of the injection route in certain cultures has not abated in the decades since the publication of,^3^^,^^30^, ^31^, ^32^ despite estimations that unsafe injections may cause millions of infections in low and middle income countries.^32^ Our study participants who preferred injections did so despite perceiving them to be painful. According to Reeler,^3^ the pain associated with injection may be perceived as a sign of a powerful medicine in many cultures. Indeed, injections received the highest efficacy scores by those who preferred injections as well as by the whole cohort. Reeler^3^ also posited how, in certain cultures, receiving injections allow recipients to feel that they have been given the best possible care, and that the injector (e.g., healthcare practitioner) ‘cares’ about their patients, which could partly explain the injection's very high acceptability score by those who prefer that route.

### 4.4 Strengths and limitations

#### 4.4.1 Strengths

This is the first study conducted in 21 countries and regions, in all cultural zones and WHO Regions, investigating the association between culture and the perceptions of 11 different routes by which medicines can be taken. The amount of missing data is low.

#### 4.4.2 Limitations

The study has some limitations. Firstly, convenience sampling was used to recruit countries where the survey would be conducted and to recruit participants. It was not feasible to ensure that the number of participants per culture region would reflect their proportion of the global population or to ensure that the genders, age groups, ethnicities, religions, health, wealth, rural vs urban locations and education levels of participants were equally represented. In this study, females, younger adults, urban dwellers, Europeans, Christians, and those with tertiary education, of average wealth, and on the healthier side were over-represented. Therefore, the results may not be fully representative of the population, although the large sample size could help to mitigate this. Secondly, the results could be influenced by the subjective measures for predictors (e.g. wealth) and outcomes (e.g. pain score). Third, due to the observational nature, the results may be confounded by unmeasured factors such as comorbidity. Another confounding factor could be the participants' experience of certain routes and their perceived efficacy and adverse effects, given that about half of the participants were taking medication. Fourth, as mentioned in the Introduction, as individuals' beliefs and values change with time, countries move across cultural classifications and maps. In this study, the WVS wave 6 (2010–2014) IW Map was used as it was the most up-to-date at the time the study was initiated. The current version of the IW map (Wave 7; 2017–2022) is slightly different to the Wave 6 map. It contains only 8 clusters (as opposed to 9 clusters in Wave 6), with the removal of the Baltic cluster. In addition, South Asia cluster has been replaced by West & South Asia cluster and six countries namely Estonia, Chile, South Africa, Kazakhstan, India and Myanmar have moved positions significantly such that they are now placed in different clusters.^33^ Most notably for this study, the Baltic cluster has been removed, and Estonia (in the Baltic cluster in Wave 6 and in this paper) is now in the Catholic Europe cluster, and India (which was in South Asia cluster in Wave 6 and in this paper) is now in the African-Islamic cluster. In this paper, the data was analysed using the Wave 6 map, as planned when the study was conducted.

We also note that while we chose the Inglehart–Welzel (IW) map, other classifications of the world have been attempted, as described by Alam,^34^ who divided the contemporary world into fourteen cultural regions, namely South Asia, East Asia, South-East Asia, West Asia, Central Asia, Russia, Europe, North Africa, Middle Africa, South Africa, North America, Middle America, South America and Australia, based on ‘a common language, presence of a strong belief in some common religion or philosophy; a social practice or a shared political or economic system or a combination of all’. Alam also notes the problems with delimitation of cultural regions of the world, the state of flux of cultures and the existence of culture border zones, rather than of sharp lines.^34^ Of particular note is the case of Turkey which is classified as African-Islamic in the Inglehart-Welzel map, but as West Asian by Alam.^34^ Other researchers have divided the Inglehart–Welzel's African-Islamic cluster into two subgroups, namely, the African cluster and the Islamic cluster.^35^ In addition to diverse classifications by researchers, individuals may also feel that their country best fits into a different category to that shown in the Inglehart-Welzel, based on, for example, geographical location or the dominant religion.

## 5 Conclusions

There is a marked association between culture and the preference for, and perceptions of the different routes by which medicines are taken. An individual's culture influenced their perception of, and preference for, even the least favoured routes (vaginal and rectal).

### 5.1 Implications

The distinct cultural element regarding the perceptions of the various routes of medicine administration and preferences for them shows the need for cultural knowledge and sensitivity, and inclusion of such factors in the education of all stakeholders involved in the medication chain such as drug developers, Heath Technology Assessment (HTA) experts, regulators and healthcare practitioners. The sparse literature about patient perceptions and preferences shows that much more research, conducted in all parts of the world, by local PIs, needs to take place in order to meet the diverse needs of all peoples. The fact that many countries are home to people with a different cultural background to the dominant one also needs to be considered. In the meantime, appropriate action needs to be taken by drug developers, researchers, healthcare practitioners and regulators when routes of medicine administration are altered. For example, currently, there is a great drive to develop non-injectable, mucosal COVID-19 vaccines, due to their potential for preventing viral infection and person-to-person transmission.^36^ Early engagement with the public to explain the reasons for the change in the route of immunization and efficacy of the new non-injectable vaccines is needed to ensure acceptance of these vaccines when they are available. Where reasonably possible, multiple routes of administration to serve people with various preferences and cultural backgrounds should also be considered for the benefit of all.

## Disclaimer

The views represented in this paper are those of the authors' and do not necessarily reflect the views and opinons of the authors' organisations, nor any of their committees or working parties.

## Declaration of Competing Interest

Authors declare that they have no conflict of interest.

## Appendix A Supplementary data

Questionnaire used in study Image 1
