# Supplementary material for: Association between culture and the preference for, and perceptions of, 11 routes of medicine administration: A survey in 21 countries and regions
Source: Explor Res Clin Soc Pharm. 2023 Nov 26;12:100378. doi: 10.1016/j.rcsop.2023.100378 (PMC10716026; doi:10.1016/j.rcsop.2023.100378)
Supplement: Supplementary file 1 — Questionnaire used in study [file mmc1.docx]

Date…..……….Country……………..Location ……………………..Questionnaire number……Initials of interviewer………..

**Taking Medicines.** When we are ill, we often have to take medicines. This survey – about the different ways of taking medicines for example, an injection, cream – is being conducted to find out what people’s views and preferences are. Please score your answers from 1 (low) to 10 (high) about the following:

**Pain/Discomfort:** How painful / uncomfortable is it to take medicines by this route?

**Efficacy:** How well does a medicine work when given by this route?

**Speed of Action:** How quickly does a medicine work when given by this route?

**Acceptability**: If your doctor prescribed a medicine, how willing would you be to take a medicine via this route?

If you have never taken medicines in these ways, score your answers by guessing how you might feel

| How do you feel when / if | Pain/Discomfort  None=1……..10= a lot | Efficacy  Not at all=1……10=Very | Speed of Action  Slow=1…….10=Quickly | Acceptability  Not at all=1….10=Very |
| --- | --- | --- | --- | --- |
| Medicine is swallowed by mouth |  |  |  |  |
| Medicine is sucked or chewed in the mouth |  |  |  |  |
| Medicine placed under the tongue |  |  |  |  |
| Drops or spray into the nose |  |  |  |  |
| Eye drops or eye ointment into the eyes |  |  |  |  |
| Ear drops into the ear |  |  |  |  |
| Breathe in a medicine into the lungs |  |  |  |  |
| Medicine injected into the body using a needle |  |  |  |  |
| Medicine applied on the skin |  |  |  |  |
| Medicine inserted into the anus/rectum |  |  |  |  |
| Medicine inserted into the vagina. Not for males |  |  |  |  |

If you could choose one of the ways above, which would you choose? ………………………………..

**Please complete the following sections with your personal information:**

Year of birth:_________

Sex:  Male  Female

Where do you live:  City  Town  Village  Countryside  Other

Ethnicity:  European  Asian  African  Arabic  Chinese  Korean  Other Please specify

Religion:  Christian  Muslim  Buddhist  Hindu  Jewish  African Traditional Religion  None  Other, Specify

How religious are you? Not at all religious 1           10 Very religious

Highest educational qualification: None  Primary School  High School  Technical Qualification

Undergraduate University degree (e.g. Bachelors)  Postgraduate University degree (e.g. Masters, Doctorate)  Other

Occupation: What is your job? ………………………………………………………………..

Wealth: In your country, do you think you are:  very rich  rich  average  poor  very poor

How would you describe your health ? Very Poor 1           10 Very Good

Are you taking any medicines?  No  Yes
